# Multi-Source Motion Inputs and FA-TA-BiLSTM for Lower-Limb Joint Angle Prediction

**DOI:** 10.3390/s26165235

**Published:** 2026-08-18

**Authors:** Chao Yang, Peng Zhao, Yuanxiang Guo, Xin Han, Xueshan Gao, Junlin Deng

**Affiliations:** 1 College of Mechanical and Marine Engineering, Beibu Gulf University, Qinzhou 535011, China; 2 School of Mechatronical Engineering, Beijing Institute of Technology, Beijing 100081, China

**Keywords:** lower-limb joint angle prediction, multi-source motion inputs, surface electromyography, bidirectional long short-term memory, feature attention, temporal attention

## Abstract

Accurate lower-limb joint angle prediction can support motion-state perception and rehabilitation-oriented analysis. This offline feasibility study evaluated an FA-TA-BiLSTM model using surface electromyography (sEMG) features, together with historical hip and knee joint angles and angular velocities. Data were collected from five healthy adult male participants during level walking and sit-to-stand transitions, and the prediction horizon was 100 ms. Support vector regression (SVR), BiLSTM, and FA-TA-BiLSTM were compared under the same combined-input condition and evaluation protocol. The FA-TA-BiLSTM model achieved RMSE, MAE, and $R^{2}$ values of 2.0684°, 1.5920°, and 0.9726 for hip prediction and 2.9604°, 2.5142°, and 0.9660 for knee prediction, respectively. These values were obtained using a mixed-participant chronological split and should be interpreted as preliminary within-cohort estimates rather than evidence of participant-independent generalization. Under this restricted protocol, FA-TA-BiLSTM produced lower errors than SVR and standard BiLSTM. The participant-level model ranking was consistent across the five participants, but exact pairwise comparisons did not reach significance after Holm adjustment. The current comparison does not isolate the incremental contribution of sEMG or individual attention modules; larger and more diverse cohorts, participant-independent validation, modality and module ablation, and causal online evaluation remain necessary.

## 1. Introduction

Lower-limb robotic and electromechanical systems have been investigated for gait rehabilitation and mobility assistance [1,2,3,4]. Accurate perception of lower-limb motion state is important for movement assessment, assisted training, and motion-intention analysis. Continuous estimation or prediction of hip and knee joint angles can provide temporal information for lower-limb motion analysis and related rehabilitation applications [5,6,7].

Human lower-limb movement is jointly affected by neuromuscular activation, joint posture, and motion dynamics. Surface electromyography (sEMG) signals reflect muscle electrical activity under neural drive and are closely related to voluntary movement intention [8,9]. However, sEMG signals are weak, nonstationary, and sensitive to electrode contact, skin impedance, motion artifacts, power-line interference, muscle fatigue, and crosstalk [10,11]. Joint angles and angular velocities provide stable kinematic descriptions but mainly represent the current motion state. Related lower-limb studies have also shown that prediction performance varies with movement conditions, sensor configurations, and participant characteristics [5,6]. Combining physiological and kinematic inputs may therefore provide a broader description of lower-limb motion than either source alone.

In the present short-horizon setting, the joint angles and angular velocities are historical observations available only up to the end of the current input sequence; they are not the future target angles. Accordingly, this study addresses short-horizon state forecasting rather than biosignal-only kinematic decoding. Historical kinematics provide autoregressive state and motion-trend cues that are already available from wearable joint sensors, whereas sEMG provides complementary physiological information associated with neuromuscular activation. Predicting the joint state 100 ms ahead may provide advance information for future delay compensation, feedforward planning, or supervisory control, although these applications were not evaluated online. Sensor-fusion and multimodal-interaction studies provide broader context for combining physiological and kinematic measurements [12,13], while multisensor and multichannel physiological sensing has been used in motion-intention recognition, locomotion-phase recognition, and continuous lower-limb analysis [14,15]. These studies support combined sensor inputs for joint angle forecasting but do not establish the independent gain of each modality in the present task.

Support vector regression (SVR) provides a conventional nonlinear regression baseline [16,17], whereas LSTM and BiLSTM networks model temporal dependencies in sequential data [18,19]. Deep learning methods have also been applied to physiological and kinematic signal analysis [20,21]. Transformer-based models, temporal convolutional networks (TCNs), and other attention-based architectures are additional sequence-learning options [22,23]. Attention mechanisms can reweight feature dimensions and temporal states, but their independent benefit requires module-level comparison.

This study evaluates an FA-TA-BiLSTM model for 100 ms-ahead hip and knee joint angle prediction using sEMG features, joint angles, and angular velocities available up to the current window. SVR and standard BiLSTM were selected as representative conventional regression and recurrent temporal baselines. The comparison is an initial offline evaluation and is not intended as a comprehensive benchmark against TCN-based, Transformer-based, or other advanced sequence models.

The methodological contributions are threefold: (1) formulation of a 100 ms-ahead lower-limb state-forecasting problem in which all physiological and kinematic inputs precede the prediction target; (2) construction of a 40-dimensional historical sequence and integration of feature-attention weighting, BiLSTM-based temporal encoding, and temporal-attention aggregation; and (3) simultaneous two-output prediction of future hip and knee joint angles using one unified model. The comparison with SVR and standard BiLSTM is used to characterize the complete framework under the present protocol rather than being presented as a separate scientific contribution. The present design does not quantify modality-specific or attention-module-specific gains.

The remainder of this paper is organized as follows. Section 2 introduces the participants, acquisition system, experimental protocol, signal processing, input construction, prediction models, and evaluation metrics. Section 3 presents the prediction results. Section 4 discusses performance, scope, and limitations. Section 5 concludes the paper.

## 2. Materials and Methods

### 2.1. Overall Framework

**Figure 1:**
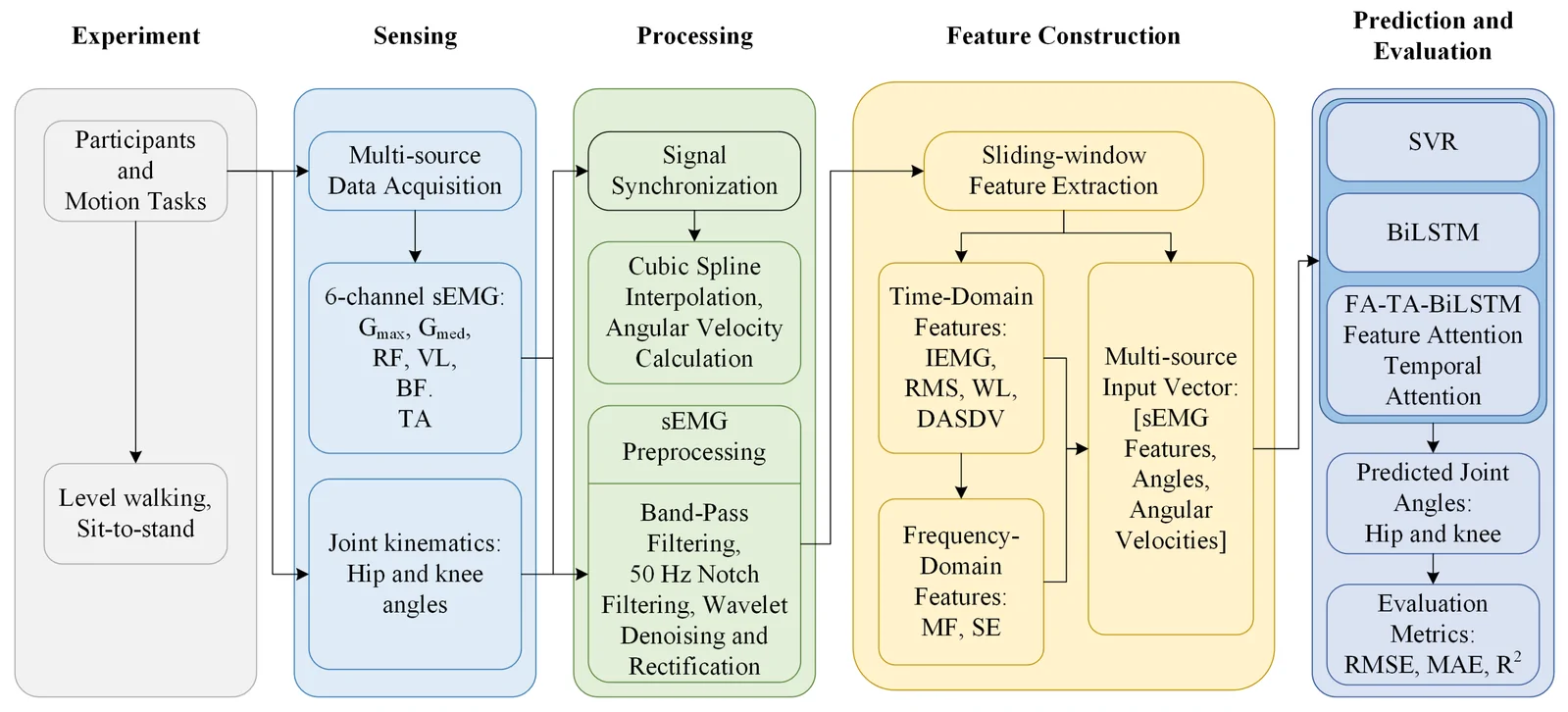
Overall framework of the proposed lower-limb joint angle prediction method.

This study establishes a lower-limb joint angle prediction framework based on combined physiological and kinematic inputs. As shown in Figure 1, the workflow consists of multi-source data acquisition, temporal synchronization, sEMG preprocessing, feature extraction, input sequence construction, model training, and performance evaluation. During data acquisition, sEMG signals are recorded from selected lower-limb muscles, while hip and knee joint angles are obtained from the inertial measurement unit (IMU)-based joint motion acquisition module. Joint angular velocities are then calculated from the synchronized angle sequences.

After synchronization, the raw sEMG signals are processed by filtering, wavelet denoising, and full-wave rectification. Time-domain and frequency-domain features are extracted using sliding windows. These sEMG features are then combined with the hip and knee joint angles and the corresponding angular velocities available up to the current feature window. The model predicts the future hip and knee joint angles over a short horizon. Under the same prediction setting and evaluation protocol, SVR, BiLSTM, and FA-TA-BiLSTM models are constructed and compared using RMSE, MAE, and $R^{2}$.

### 2.2. Participants

Five healthy adult male participants took part in the experiment. Eligibility was assessed by verbal pre-experiment screening for self-reported lower-limb musculoskeletal injury, neuromuscular disease, obvious gait abnormality, or other conditions that could affect task performance; no formal medical examination or diagnostic assessment was performed. To reduce short-term fatigue, participants were asked not to perform strenuous exercise within 12 h before the experiment. The purpose, movement requirements, and safety precautions were explained, and written informed consent was obtained. This small homogeneous cohort was used for a preliminary within-cohort offline evaluation and does not represent broader demographic or clinical populations. The participant count was determined by feasibility and participant availability rather than by an a priori power calculation. The participant, rather than the large number of overlapping windows, is the independent biological unit; therefore, the present sample is insufficient for confirmatory population-level inference. Participant characteristics are summarized in Table 1.

### 2.3. Multi-Source Sensor Acquisition System

#### 2.3.1. Sensor Configuration

The acquisition system comprised an sEMG module and an IMU-based joint-motion module. Both were existing laboratory systems purchased and configured before the present study. Because the original procurement records were unavailable, the exact manufacturer and commercial model information could not be reliably verified; only the confirmed configuration and acquisition parameters are reported.

**Figure 2:**
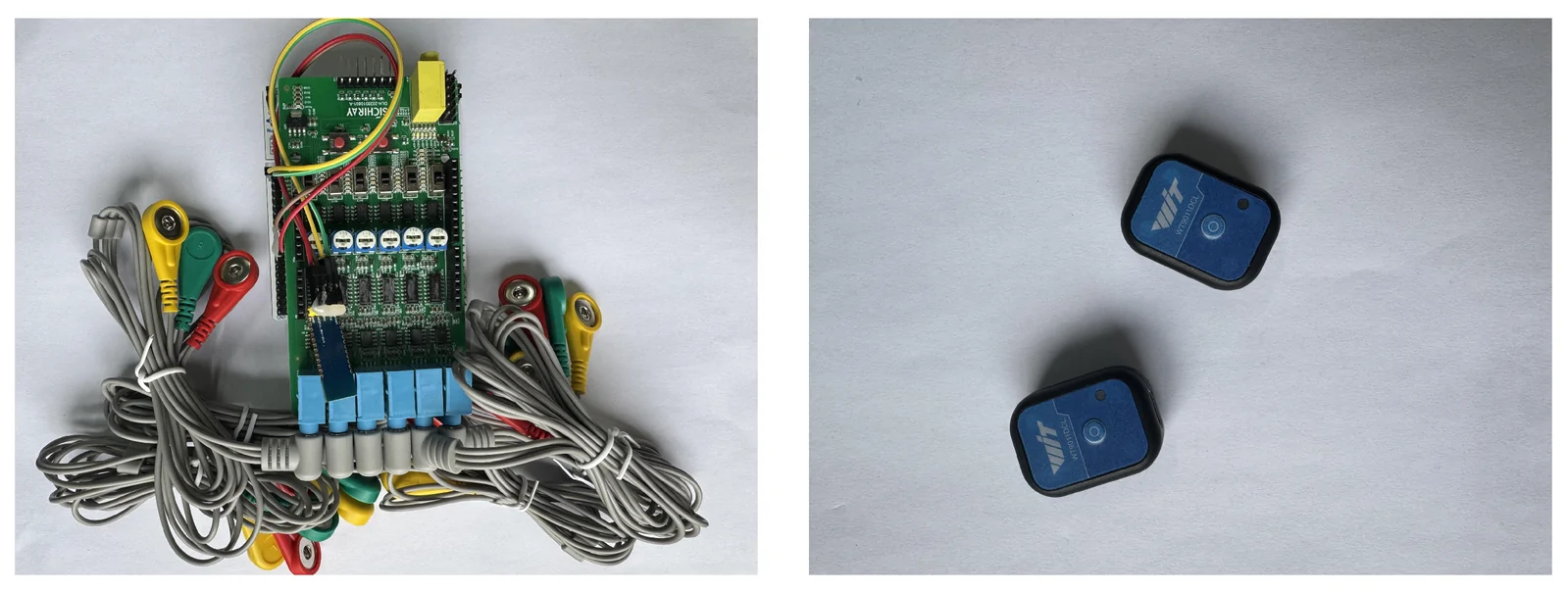
Hardware architecture of the multi-source acquisition system.

The sEMG signals were recorded at 1000 Hz, whereas the IMU-based joint-motion signals were recorded at 200 Hz. The two systems were manually started as close in time as possible and did not share a hardware trigger or clock. Their returned timestamps were subsequently used for software-level alignment. sEMG provided complementary neuromuscular information, while joint angles and angular velocities described posture and motion trend. Their separate contributions were not independently quantified. The functional roles of the input sources are summarized in Table 2, and the acquisition hardware is shown in Figure 2.

#### 2.3.2. Muscle Selection and Electrode Placement

The choice of target muscles affects how well the sEMG signals represent lower-limb motor intent and joint motion. Because the prediction targets are the hip and knee joint angles, the selected muscles should cover hip actuation, knee flexion and extension, pelvic control, and gait-timing-related functions. An insufficient number of muscles may lead to incomplete motion-related information, whereas excessive channels may increase acquisition complexity and introduce redundancy or muscle crosstalk [24,25].

Considering anatomical accessibility, functional relevance, electrode placement stability, and hip–knee coordination, six muscles were selected as sEMG acquisition targets: gluteus maximus, gluteus medius, rectus femoris, vastus lateralis, biceps femoris, and tibialis anterior. The gluteus maximus is mainly involved in hip extension and reflects support, propulsion, and trunk lifting. The gluteus medius contributes to pelvic stabilization and hip control. The rectus femoris crosses both the hip and knee joints and participates in hip flexion and knee extension. The vastus lateralis mainly reflects knee extensor activity. The biceps femoris participates in knee flexion and assists hip extension. Although the tibialis anterior does not directly actuate the hip or knee joint, its activity is closely related to foot control during the swing phase and can provide useful temporal information for lower-limb motion prediction [24,26]. The functions of the six target muscles are summarized in Table 3.

**Figure 3:**
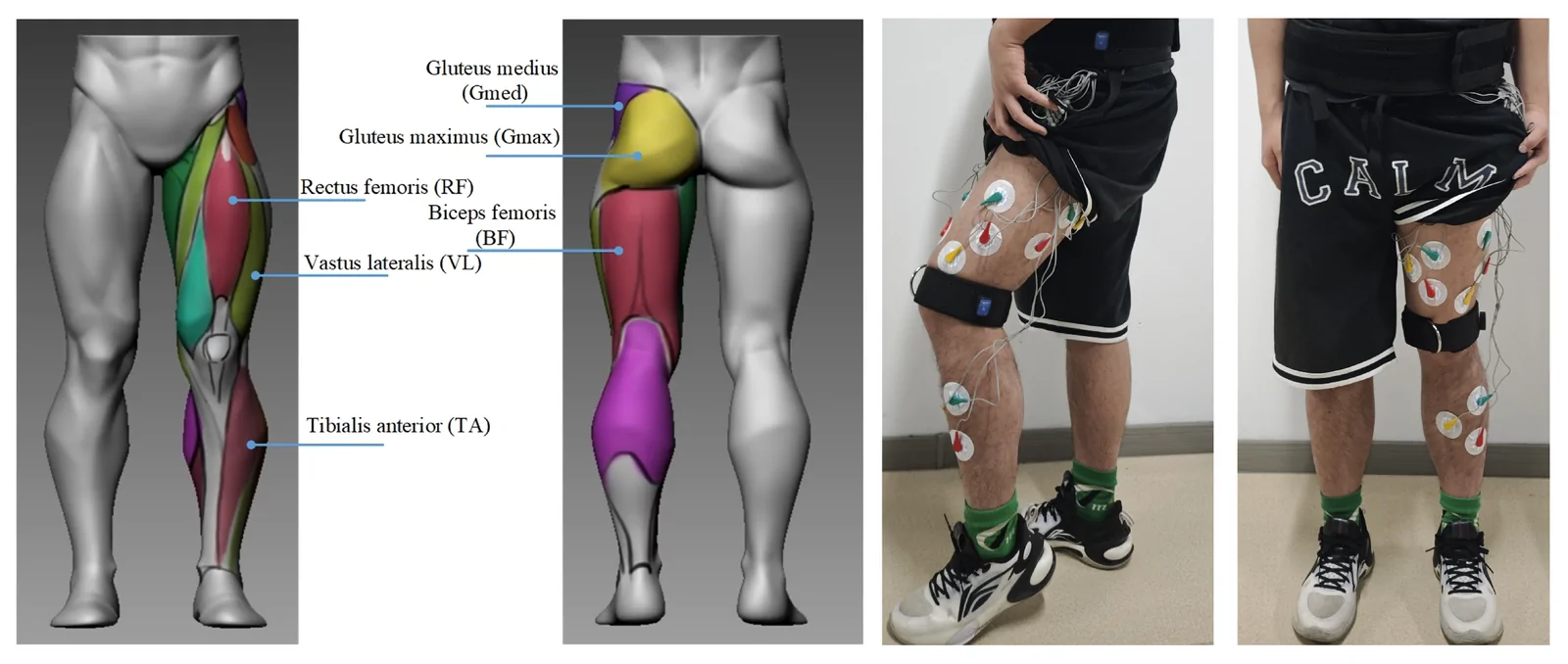
Surface distribution of target muscles and electrode placement.

Before electrode placement, the target muscle regions were identified, and the skin surface was cleaned to reduce the influence of skin impedance. Electrodes were placed away from tendon regions, bony prominences, and areas where adjacent muscles overlapped as much as possible. For each participant, electrode positions were kept consistent across different trials to improve the repeatability of the collected sEMG data [25,27]. The target-muscle distribution and electrode locations are illustrated in Figure 3.

### 2.4. Experimental Protocol

Two offline motion-acquisition tasks were used: level walking and sit-to-stand transitions. During the level-walking task, participants walked on level ground at a natural, self-selected speed. This task provided continuous, approximately periodic lower-limb motion data. During the transition task, participants moved from standing to sitting and then returned to standing. This task represented a common nonperiodic postural transition.

**Figure 4:**
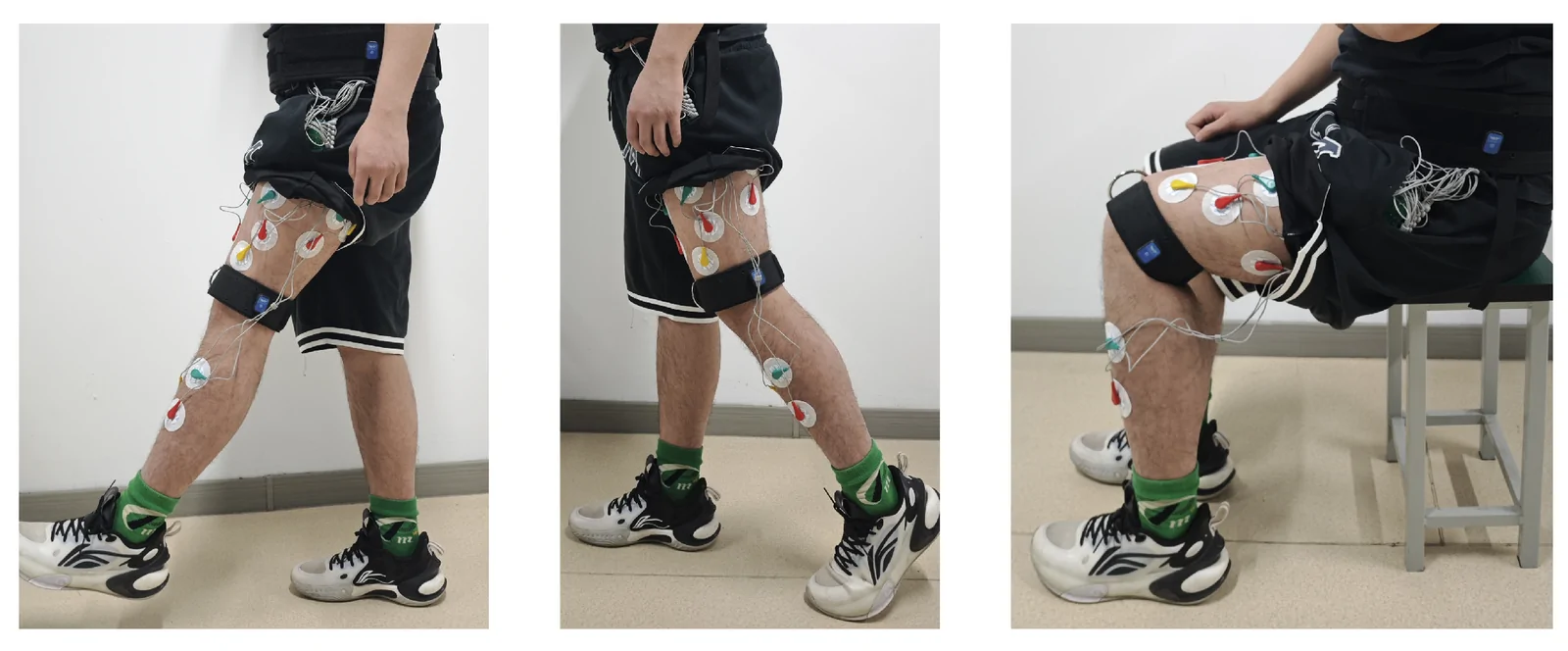
Schematic of the experimental motion tasks.

One trial was defined as one continuous recording of a specified movement task rather than one gait cycle or one sit-to-stand cycle. Each participant completed 10 level-walking trials and 10 sit-to-stand trials, yielding 20 trials per participant, and each trial lasted at least 30 s. To reduce fatigue, a 5 min rest period was provided after every five trials. Participants were instructed to move consistently and avoid sudden acceleration, deceleration, or excessive trunk sway. For each participant, the valid recordings from both tasks were concatenated in their stored order before sliding-window segmentation. Consequently, windows and 16-window input sequences located near recording or task junctions may contain samples from adjacent segments. Task- and trial-boundary identifiers were not retained in the processed dataset, so these boundary samples could not be isolated retrospectively. The samples from both tasks were used to train a single prediction model, and the reported metrics therefore describe aggregate performance across both tasks rather than task-specific results. For each sample, a 200 ms input window and a 50 ms step were used, and the hip and knee angles 100 ms after the input window were the prediction targets. The two motion tasks are illustrated in Figure 4.

### 2.5. Signal Synchronization and Preprocessing

#### 2.5.1. Signal Synchronization

After alignment by the returned timestamps, the 1000 Hz sEMG timeline was used as the reference and the 200 Hz IMU-derived hip and knee angle sequences were resampled by cubic spline interpolation. The interpolation was used only for temporal alignment; it did not increase the original temporal information and may introduce limited smoothing or local overshoot during rapid angle changes. The synchronized joint angle sequence can be written as follows:

(1) $$\theta_{s}\left(t_{k}\right)=S\left(t_{k}\right).$$

where S(t) is the interpolation function constructed from the original joint angle samples, and $t_{k}$ is the sampling instant on the unified time axis. Based on the synchronized joint angle sequence, joint angular velocity in rad/s is calculated from angles in degrees as follows:

(2) $$\omega_{j}\left(t_{k}\right)=\frac{\pi }{180}\;\frac{\theta_{s,j}\left(t_{k}\right)-\theta_{s,j}\left(t_{k-1}\right)}{\Delta t},\;j\in \left\{\mathrm{hip},\mathrm{knee}\right\}.$$

**Figure 5:**
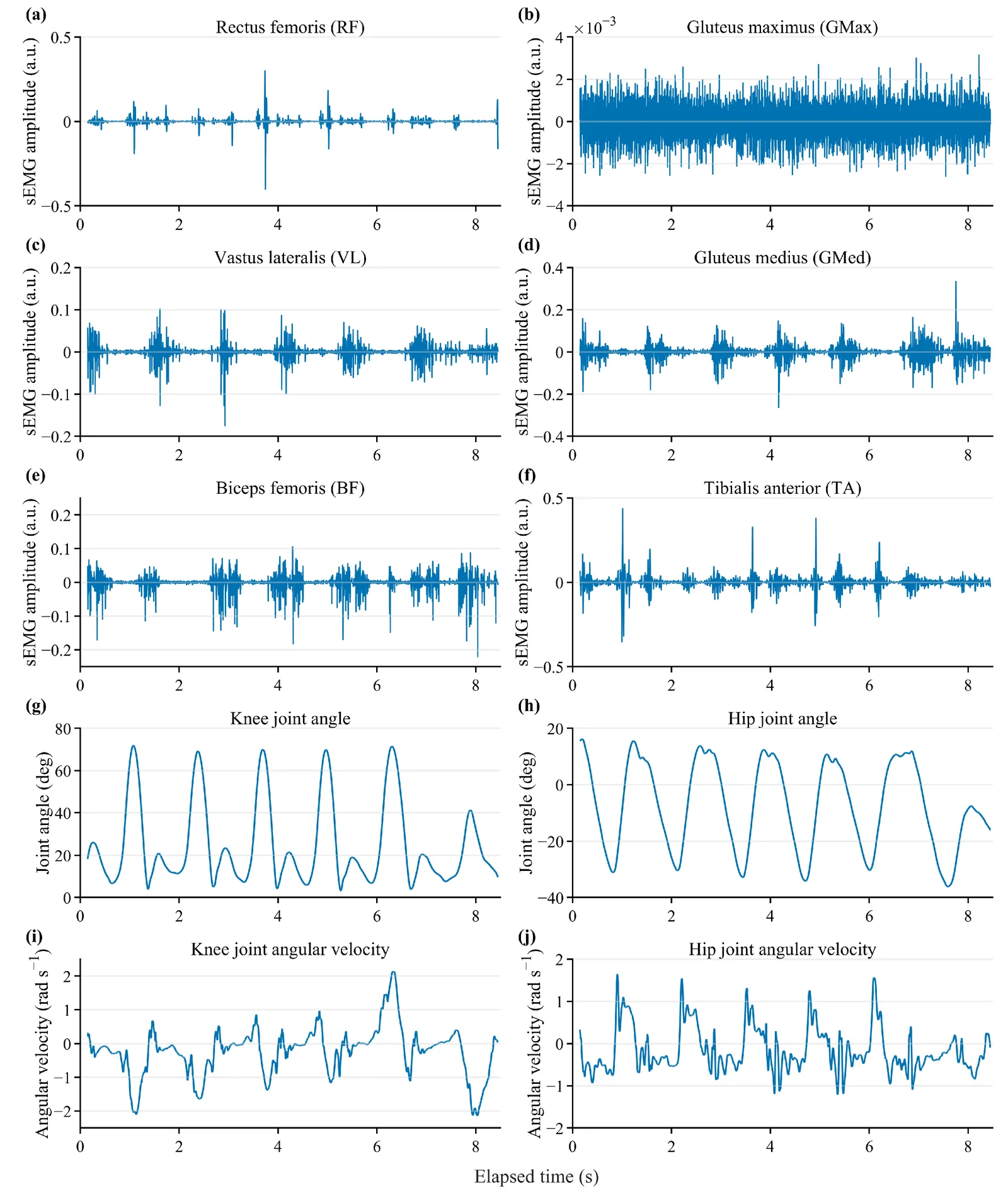
Synchronization results for six-channel sEMG, hip and knee joint angles, and the corresponding hip and knee joint angular velocities: (**a**) rectus femoris (RF); (**b**) gluteus maximus (GMax); (**c**) vastus lateralis (VL); (**d**) gluteus medius (GMed); (**e**) biceps femoris (BF); (**f**) tibialis anterior (TA); (**g**) knee joint angle; (**h**) hip joint angle; (**i**) knee joint angular velocity; and (**j**) hip joint angular velocity.

Through this processing, sEMG features, joint angles, and angular velocities were matched at the feature-window level. The angular velocity was derived from the synchronized angle sequence and should be interpreted as a kinematic derivative rather than an independently measured high-frequency signal. The synchronized IMU-derived angles served as the common measured references for training and evaluation of all models. No independent validation against optical motion capture or a goniometer was performed; accordingly, the reported errors are relative to the IMU measurements rather than absolute anatomical-angle accuracy. Residual timing uncertainty from manual starting and independent clocks cannot be excluded. The synchronized signals are shown in Figure 5.

#### 2.5.2. sEMG Preprocessing

Raw sEMG signals can be affected by motion artifacts, power-line interference, random noise, and electrode-contact variation [11]. After channel-wise mean removal, a fourth-order zero-phase Butterworth bandpass filter of 20–450 Hz and a 50 Hz notch filter with a quality factor of 35 were applied [28].

Wavelet denoising was then applied because sEMG is nonstationary [29,30]. MATLAB R2023b wdenoise was used with five decomposition levels and the Daubechies 4 (db4) wavelet; all unspecified denoising options retained their software-default values. The denoising process can be expressed as follows:

(3) $$x_{2}\left(n\right)=W^{-1}\left(T\left(W\left(x_{1}\left(n\right)\right)\right)\right).$$

where $x_{1}\left(n\right)$ is the signal after bandpass and notch filtering, $x_{2}\left(n\right)$ is the denoised signal, W(·) and $W^{-1}\left(\cdot \right)$ denote wavelet decomposition and reconstruction, and T(·) denotes the automatic thresholding operation. Finally, full-wave rectification was used to transform the bipolar sEMG waveform into an amplitude-related signal:

(4) $$x_{3}\left(n\right)=\left|x_{2}\left(n\right)\right|.$$

**Figure 6:**
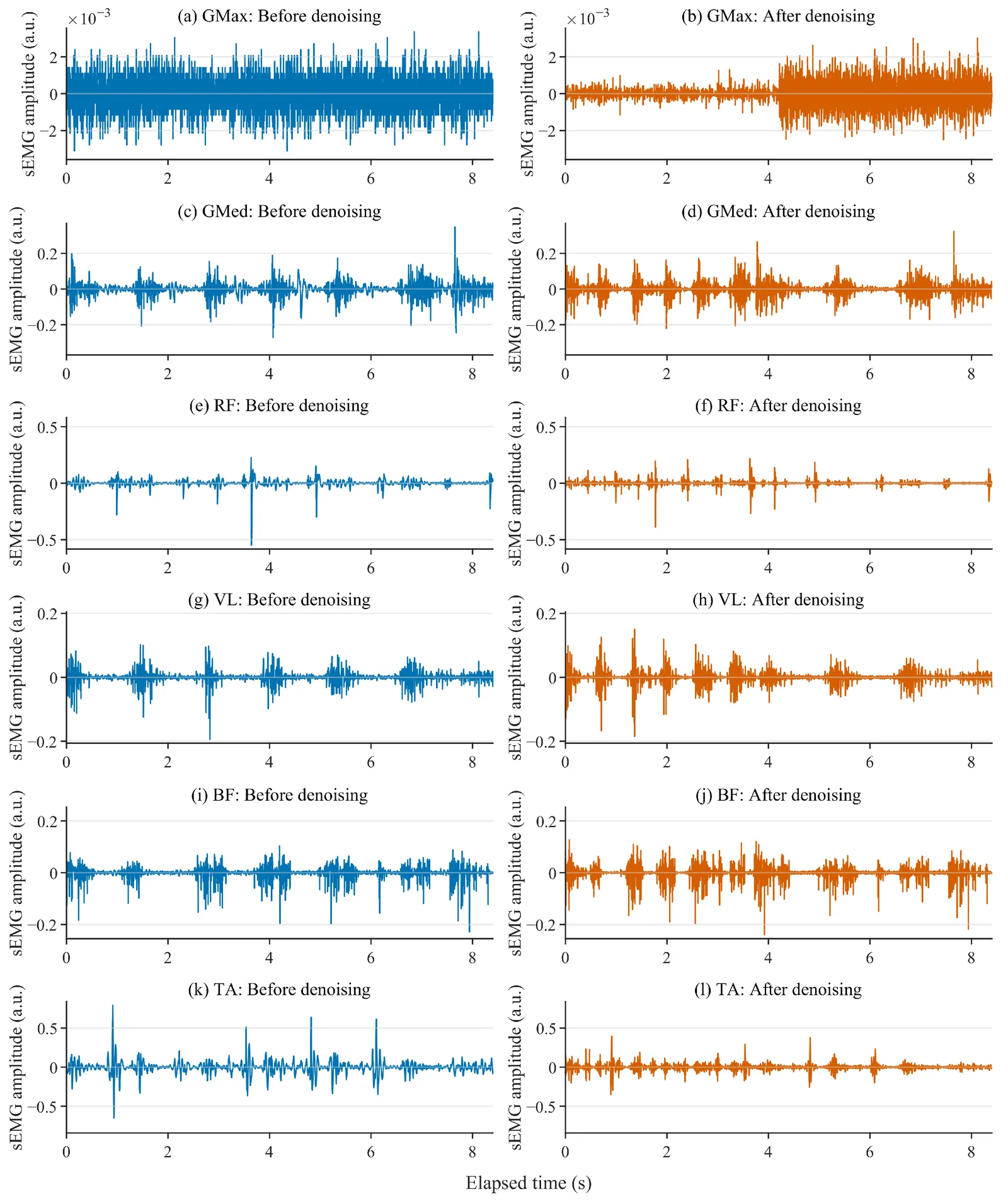
Comparison of sEMG signals before and after denoising.

After full-wave rectification, the signals were segmented directly for feature extraction; no moving-average, RMS-envelope, or low-pass smoothing was applied. This procedure reduced major interference components while retaining amplitude variations related to muscle activation. Figure 6 shows illustrative sEMG waveforms before and after denoising.

### 2.6. Feature Extraction and Multi-Source Input Construction

#### 2.6.1. sEMG Feature Extraction

After preprocessing, continuous sEMG signals were segmented using a sliding window. The window length was set to 200 ms, and the window step was set to 50 ms. This study used this fixed window configuration to balance local feature estimation and temporal sampling density. For the *i*th window, the sEMG samples are denoted as follows:

(5) $$X_{i}=\left\{x\left(\left(i-1\right)S+1\right),x\left(\left(i-1\right)S+2\right),\ldots ,x\left(\left(i-1\right)S+L\right)\right\},\;i=1,2,\ldots .$$

where *L* is the window length and *S* is the window step. For each sEMG channel, four time-domain features and two frequency-domain features were extracted, namely IEMG, RMS, WL, DASDV, MF, and SE. Time-domain descriptors, including waveform-length-based measures, have a long history in myoelectric feature construction [10,31]. These features describe amplitude accumulation, signal energy, waveform variation, local fluctuation, spectral energy distribution, and spectral complexity. Their definitions are summarized in Table 4.

In Table 4, x(k) is the *k*th sample in a window, *N* is the number of samples, P(f) is the power spectral density, $f_{\mathrm{MF}}$ is the median frequency corresponding to half of the total spectral power, $p_{j}$ is the normalized spectral power component, and *M* is the number of frequency components. The natural logarithm is used in the SE calculation. The six features extracted from each of the six sEMG channels were concatenated in a fixed order to form the sEMG feature vector $F_{emg}\left(i\right)$. These features describe activation amplitude, signal energy, waveform variation, frequency distribution, and spectral complexity. Some features may contain overlapping information; for example, IEMG and RMS are both amplitude-related, while WL and DASDV both describe waveform variation. The 36-dimensional sEMG vector was retained as a fixed representation, and no feature-selection or feature-importance analysis was performed. The learned attention representations should therefore not be interpreted as direct physiological importance scores for individual raw features.

#### 2.6.2. Multi-Source Input Construction

Table 5 summarizes the composition and dimensionality of the combined input. After feature extraction and synchronization, the sEMG features, joint angles, and joint angular velocities were integrated into a unified input vector:

(6) $$X\left(i\right)=[F_{emg}\left(i\right),F_{ang}\left(i\right),F_{vel}\left(i\right)].$$

where $F_{emg}\left(i\right)$ denotes the sEMG feature vector, $F_{ang}\left(i\right)$ contains the synchronized hip and knee joint angles, and $F_{vel}\left(i\right)$ contains the corresponding angular velocities. All variables in X(i) are information available at or before the current feature window. The model does not use the joint angles of the current window as the prediction labels. Instead, the target output contains the future hip and knee joint angles after a fixed prediction horizon.

Given the historical input sequence {X(i−T+1),…,X(i)}, the model predicts the future joint angle vector as

(7) $$\hat{y}\left(i+h\right)=f\left(\left\{X\left(i-T+1\right),\ldots ,X\left(i\right)\right\}\right),\;y\left(i+h\right)={\left[\theta_{s,\mathrm{hip}}\left(t_{i}+\Delta t_{p}\right),\theta_{s,\mathrm{knee}}\left(t_{i}+\Delta t_{p}\right)\right]}^{T}.$$

where *T* is the input sequence length, *h* is the label shift in feature steps, $t_{i}$ denotes the end time of the current feature window, and $\Delta t_{p}$ is the prediction horizon. In this study, $\Delta t_{p}$ was set to 100 ms. Since the feature window step was 50 ms, the target labels were shifted by two feature steps relative to the last input window. Therefore, the current joint angles and angular velocities were used as available state variables for short-horizon forecasting, rather than as the output labels of the same time window. The complete prediction configuration is summarized in Table 6.

Since different input variables have different units and numerical ranges, min–max normalization was applied before model training [32]:

(8) $$x^{\prime }=\frac{x-x_{\min}}{x_{\max}-x_{\min}}.$$

where *x* is the original value, $x^{\prime }$ is the normalized value, and $x_{\min}$ and $x_{\max}$ are the minimum and maximum values of the corresponding feature calculated from the training set. The same normalization parameters were then applied to the validation and test sets.

### 2.7. Prediction Models

Three models were compared under the same dataset division, feature set, prediction horizon, and evaluation metrics. SVR was used as a conventional nonlinear regression baseline. BiLSTM was used to model temporal dependencies in the multi-source sequence. FA-TA-BiLSTM further introduced feature attention and temporal attention to reweight input dimensions and historical hidden states, respectively. The comparison in this study evaluates the overall performance of the complete FA-TA-BiLSTM architecture under the constructed multi-source input condition. It is not intended to separately quantify the independent contribution of each sensor modality or each attention module, nor should it be interpreted as a comprehensive comparison with TCN, Transformer-based, or other advanced sequence models.

#### 2.7.1. Support Vector Regression

Support vector regression maps input samples into a high-dimensional feature space and fits nonlinear relationships between input features and target joint angles [16,17]. For training samples ${\left\{\left(x_{i},y_{i}\right)\right\}}_{i=1}^{N}$, the regression function is

(9) $$f\left(x\right)=w^{T}\phi \left(x\right)+b.$$

The radial basis function kernel was adopted to describe nonlinear sample distributions with limited manual assumptions:

(10) $$K\left(x_{i},x_{j}\right)=\exp(-\gamma ∥x_{i}-x_{j}{∥}_{2}^{2}).$$

The Gaussian kernel was used for the SVR baseline. In the implementation, the penalty factor, kernel scale, and insensitive loss setting were specified in advance, with the kernel scale set to automatic selection rather than manually assigning a fixed γ value. The detailed settings are summarized later with the model training settings.

#### 2.7.2. Bidirectional Long Short-Term Memory

BiLSTM contains a forward LSTM and a backward LSTM. It can model temporal relationships from both directions within a given input sequence and is suitable for continuous lower-limb motion data with temporal dependence [18,19]. In this study, only historical input sequences up to the current feature window were provided to the model. The backward direction processed this known historical segment in reverse order, and no samples after the current window or target time were included in the input. Thus, bidirectional modeling was used for contextual encoding of an observed finite sequence rather than for accessing future targets. The evaluated model nevertheless requires the complete buffered historical sequence and is not equivalent to strictly causal online streaming. The BiLSTM output at time *t* is expressed as the concatenation of the forward and backward hidden states:

(11) $$H_{t}=\left[\vec{h_{t}},\overset{\leftarrow }{h_{t}}\right].$$

where $\vec{h_{t}}$ and $\overset{\leftarrow }{h_{t}}$ are the forward and backward hidden states, respectively. The resulting vector $H_{t}$ is used as the temporal representation of the multi-source input sequence. A unidirectional LSTM or another causal temporal model should be evaluated for real-time deployment.

#### 2.7.3. Feature- and Temporal-Attention-Based BiLSTM

**Figure 7:**
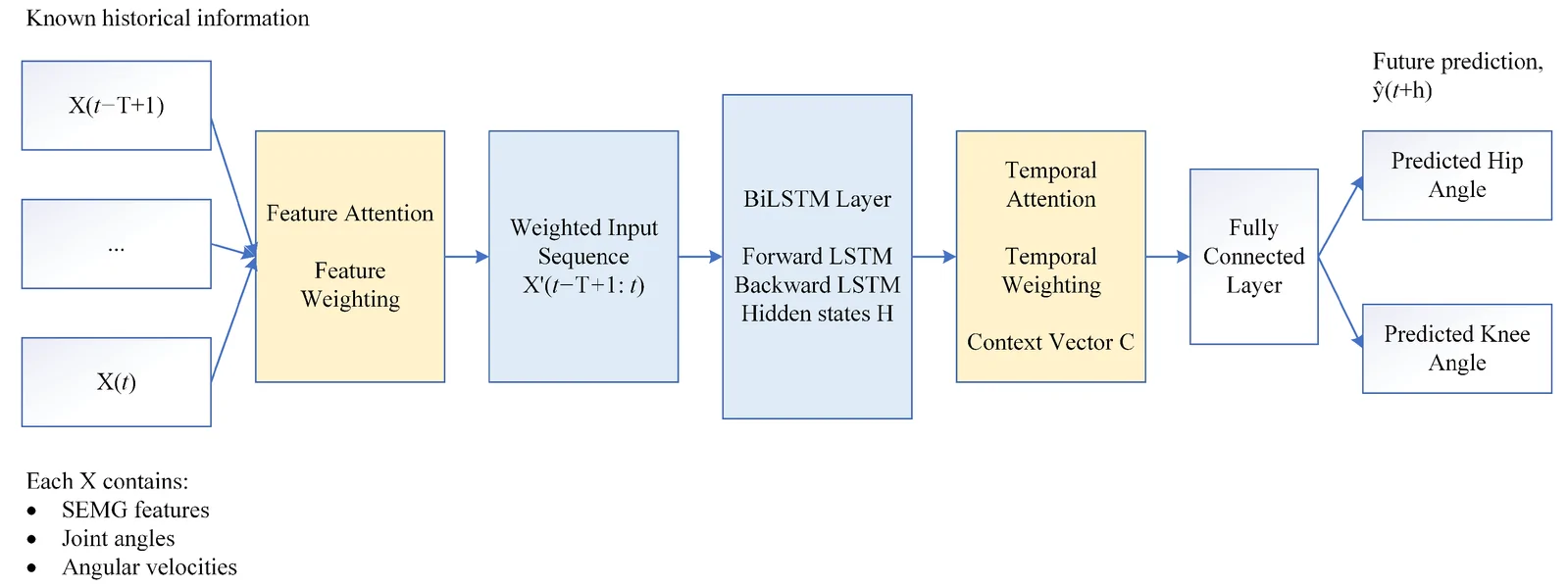
Architecture of the FA-TA-BiLSTM prediction model.

The FA-TA-BiLSTM model follows the functional flow shown in Figure 7. Feature attention first reweights the 40 input dimensions at each historical time step, allowing the model to emphasize sensor-derived variables that are more informative for the current motion state. The feature-weighted sequence is then encoded by a BiLSTM temporal encoder. Temporal attention subsequently assigns weights to the BiLSTM hidden states across the observed sequence and aggregates them into a context vector for two-output regression. The BiLSTM block in Figure 7 represents the recurrent encoder as a functional unit; its principal settings are summarized later with the model training settings. Because no module-level ablation was performed, the separate contributions of feature attention and temporal attention are not quantified in this study.

For an input sequence $X=[X_{1},\ldots ,X_{T}]$, where $X_{t}\in R^{D}$ and D=40, the feature-attention weights and the reweighted input are defined as

(12) $$\alpha_{t}=\mathrm{softmax}\;\left(W_{\mathrm{fa}}X_{t}+b_{\mathrm{fa}}\right),\;{\tilde{X}}_{t}=\alpha_{t}⊙X_{t},$$

where $\alpha_{t}\in R^{D}$ is the feature-attention weight vector, $W_{\mathrm{fa}}$ and $b_{\mathrm{fa}}$ are learnable parameters, and ⊙ denotes element-wise multiplication. The weighted sequence $\left[{\tilde{X}}_{1},\ldots ,{\tilde{X}}_{T}\right]$ is processed by the BiLSTM encoder to obtain the hidden-state sequence ${\left\{H_{t}\right\}}_{t=1}^{T}$, as defined in Equation (11).

Temporal attention evaluates the relevance of each historical hidden state to the prediction target. The attention scores, normalized temporal weights, and context vector are defined as [33]

(13) $$\begin{array}{cc} u_{t} & =\tanh\;\left(W_{\mathrm{ta}}H_{t}+b_{\mathrm{ta}}\right),\;e_{t}=v_{\mathrm{ta}}^{T}u_{t},\\ \beta_{t} & =\frac{\exp(e_{t})}{\sum_{k=1}^{T}\exp\left(e_{k}\right)},\;C=\sum_{t=1}^{T}\beta_{t}H_{t}, \end{array}$$

where $e_{t}$ is the temporal-attention score, $\beta_{t}$ is the normalized temporal weight, $W_{\mathrm{ta}}$, $b_{\mathrm{ta}}$, and $v_{\mathrm{ta}}$ are learnable parameters, and *C* is the context vector. The future hip and knee joint angles are then predicted through the regression head:

(14) $$\hat{y}=W_{c}C+b_{c}={\left[{\hat{\theta }}_{\mathrm{hip}},{\hat{\theta }}_{\mathrm{knee}}\right]}^{T}.$$

Figure 7 and Equations (12)–(14) therefore describe the same feature-attention–BiLSTM–temporal-attention information flow.

### 2.8. Model Training and Evaluation Metrics

#### 2.8.1. Experimental Settings

SVR, BiLSTM, and FA-TA-BiLSTM were evaluated using the same combined input, 100 ms prediction horizon, dataset split, and metrics. For each participant, the constructed sequence samples were divided chronologically into 70% training, 15% validation, and 15% test subsets. The five participant-specific training subsets were then concatenated to form the overall training set, and the validation and test subsets were combined in the same manner. Consequently, all five participants contributed samples to the training, validation, and test sets. This protocol provides a mixed-participant, within-cohort evaluation rather than participant-independent generalization. Because window construction preceded the split and adjacent 200 ms windows overlap by 75%, chronological division reduces random mixing but does not eliminate correlation between neighboring samples near subset boundaries. The training set was used for parameter learning, the validation set supported manual model selection and hyperparameter adjustment, and the test set was used for evaluation. The listed hyperparameters were manually selected during preliminary development with reference to validation performance and training stability; no exhaustive grid or random search was conducted.

Table 7 reports the participant-level recording durations, usable labeled-window counts, sequence-sample counts, and chronological 70/15/15 splits before the corresponding participant-specific subsets were pooled across participants. The final two raw windows of each recording stream were excluded because a 100 ms future target was unavailable.

Figure 7 and Equations (12)–(14) describe the functional information flow of the proposed model: feature-wise input reweighting, bidirectional temporal encoding, temporal aggregation, and two-output regression. The BiLSTM and FA-TA-BiLSTM models use two output neurons corresponding to the predicted hip and knee joint angles. For the SVR baseline, two separate single-output regressors were trained for hip and knee angle prediction. In all compared models, the input feature dimension was 40, the input window length was 200 ms, the sliding step was 50 ms, the prediction horizon was 100 ms, and 16 consecutive windows were used to form one input sequence. These overlapping windows span approximately 950 ms from the beginning of the first window to the end of the last window. The principal model components and training settings are summarized in Table 8.

#### 2.8.2. Evaluation Metrics

Prediction accuracy was evaluated by RMSE, MAE, and $R^{2}$ [34,35]. The metrics were calculated separately for the hip and knee output channels. RMSE emphasizes the overall error magnitude and is sensitive to large deviations. MAE reflects the average absolute deviation. $R^{2}$ describes how well the predicted sequence explains the variation in the measured joint angle. Their definitions are:

(15) $$RMSE=\sqrt{\frac{1}{N}\sum_{i=1}^{N}{\left(y_{i}-{\hat{y}}_{i}\right)}^{2}},\;MAE=\frac{1}{N}\sum_{i=1}^{N}\left|y_{i}-{\hat{y}}_{i}\right|.$$

(16) $$R^{2}=1-\frac{\sum_{i=1}^{N}{\left(y_{i}-{\hat{y}}_{i}\right)}^{2}}{\sum_{i=1}^{N}{\left(y_{i}-\bar{y}\right)}^{2}}.$$

where $y_{i}$ is the measured joint angle at the target time, ${\hat{y}}_{i}$ is the predicted joint angle, $\bar{y}$ is the mean value of measured joint angles, and *N* is the number of test samples. Lower RMSE and MAE values indicate smaller prediction errors, while an $R^{2}$ closer to 1 indicates better agreement between the predicted and measured trajectories.

#### 2.8.3. Participant-Level Statistical Analysis

The participant (N=5), rather than individual overlapping windows, was treated as the unit of statistical comparison. Per-participant RMSE and MAE values were analyzed separately for the hip and knee joints. For each of the four outcomes, a Friedman test compared the three models, and Kendall’s coefficient of concordance (*W*) was reported as an effect-size measure. When the omnibus test was significant, planned exact two-sided Wilcoxon signed-rank tests compared FA-TA-BiLSTM with SVR and with standard BiLSTM. The two planned comparisons within each outcome were adjusted using the Holm procedure. Because the cohort was small and no a priori power analysis was performed, these analyses are exploratory and are not interpreted as confirmatory evidence of population-level superiority.

## 3. Results

**Figure 8:**
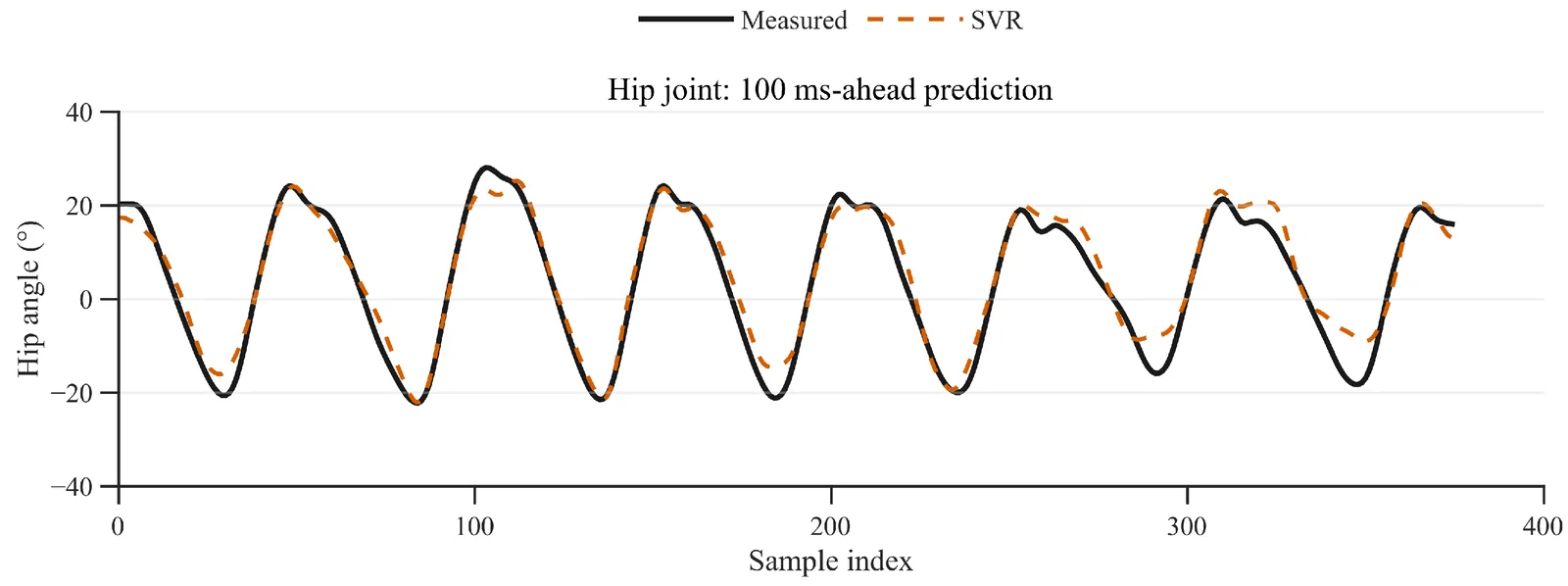
Illustrative SVR prediction and IMU-derived measured hip angle over the fixed test-output interval defined at the beginning of Section 3.

**Figure 9:**
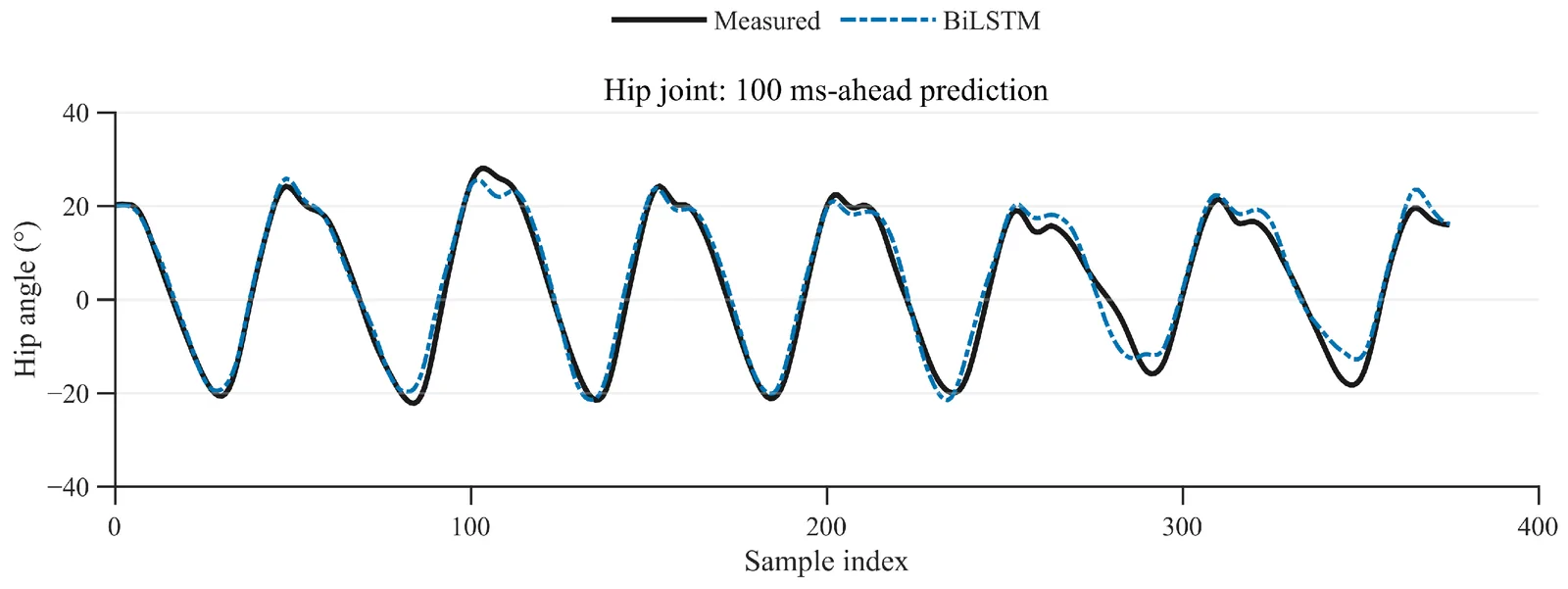
Illustrative BiLSTM prediction and IMU-derived measured hip angle over the fixed test-output interval defined at the beginning of Section 3.

**Figure 10:**
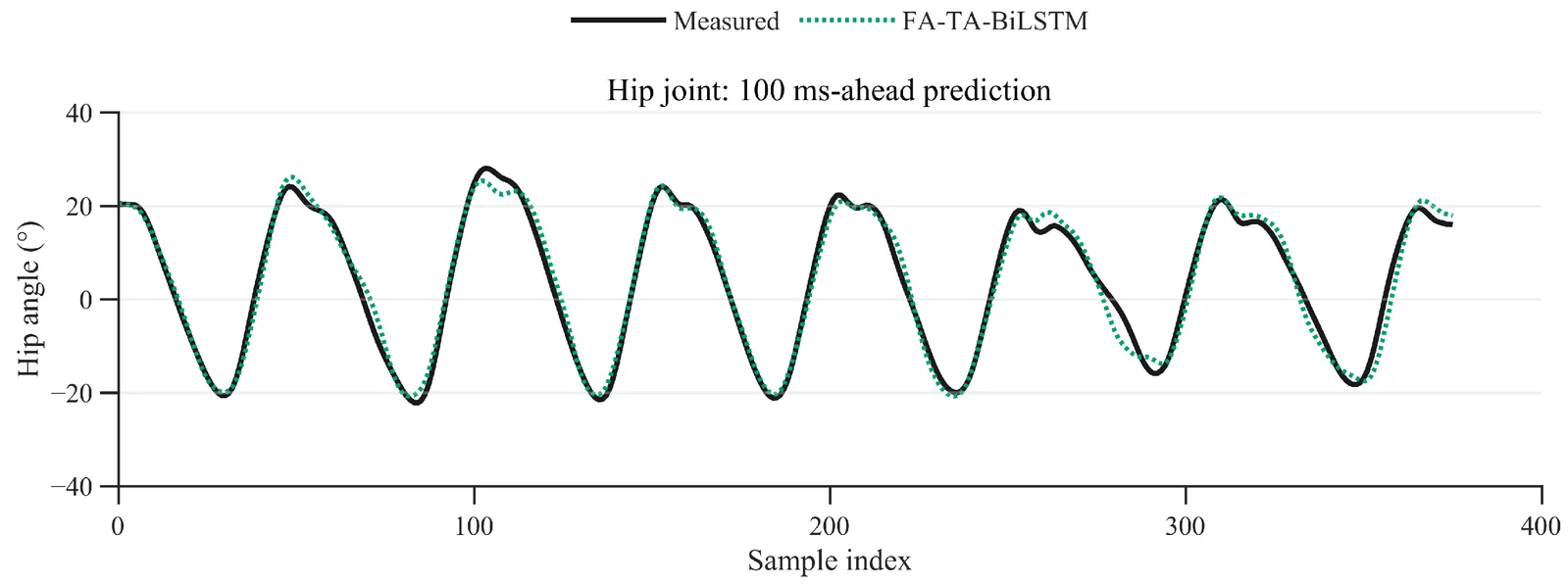
Illustrative FA-TA-BiLSTM prediction and IMU-derived measured hip angle over the fixed test-output interval defined at the beginning of Section 3.

**Figure 11:**
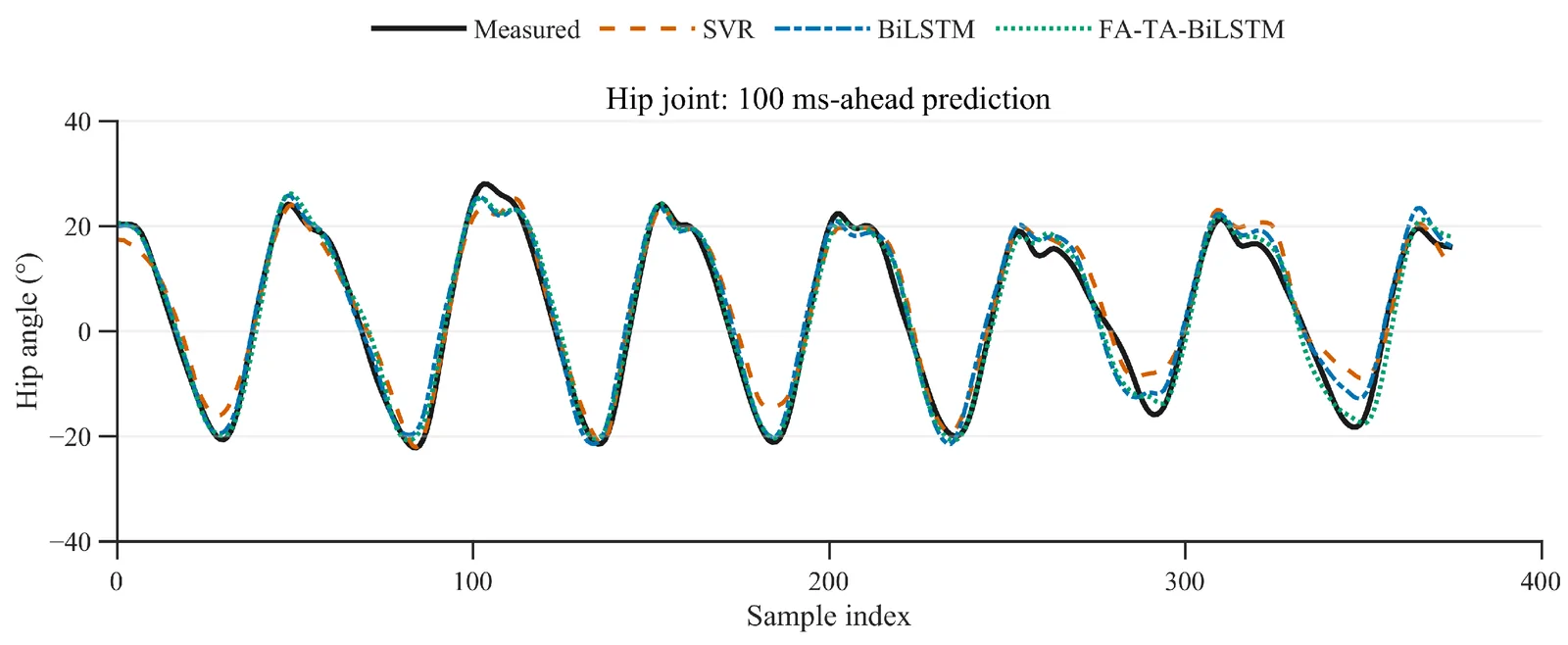
Illustrative comparison of hip angle predictions from the three models against the IMU-derived reference over the fixed test-output interval.

**Figure 12:**
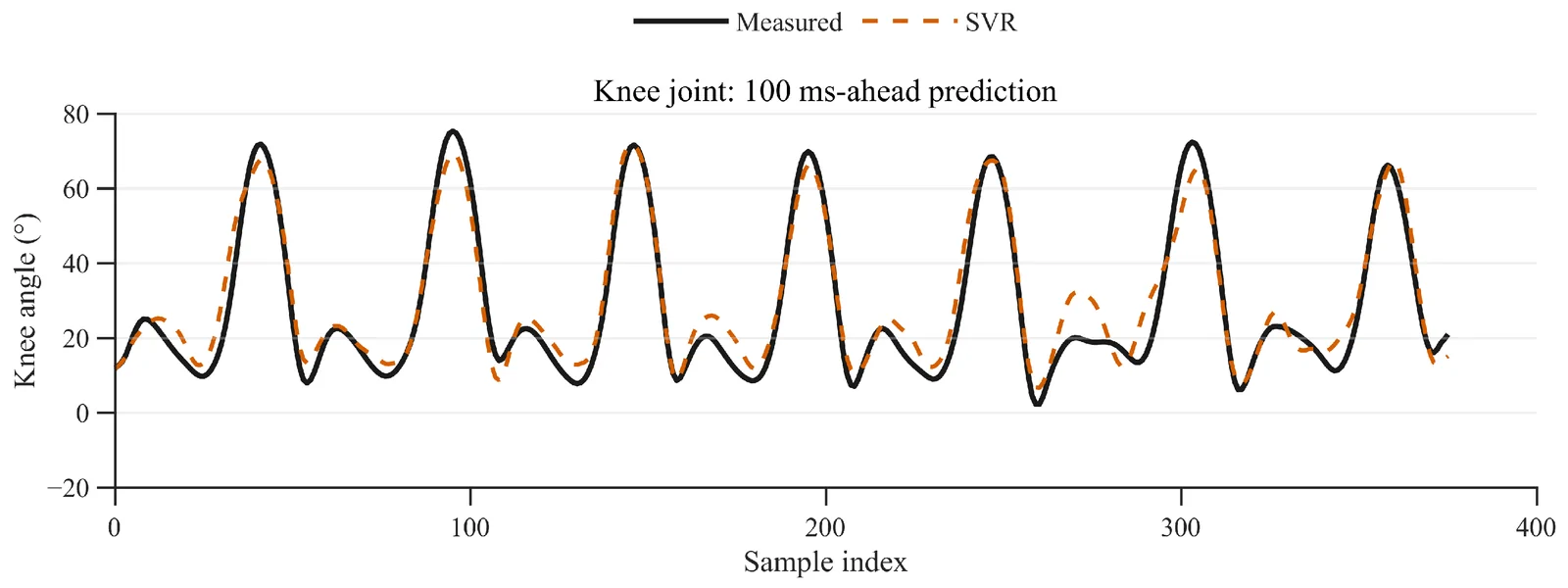
Illustrative SVR prediction and IMU-derived measured knee angle over the fixed test-output interval defined at the beginning of Section 3.

**Figure 13:**
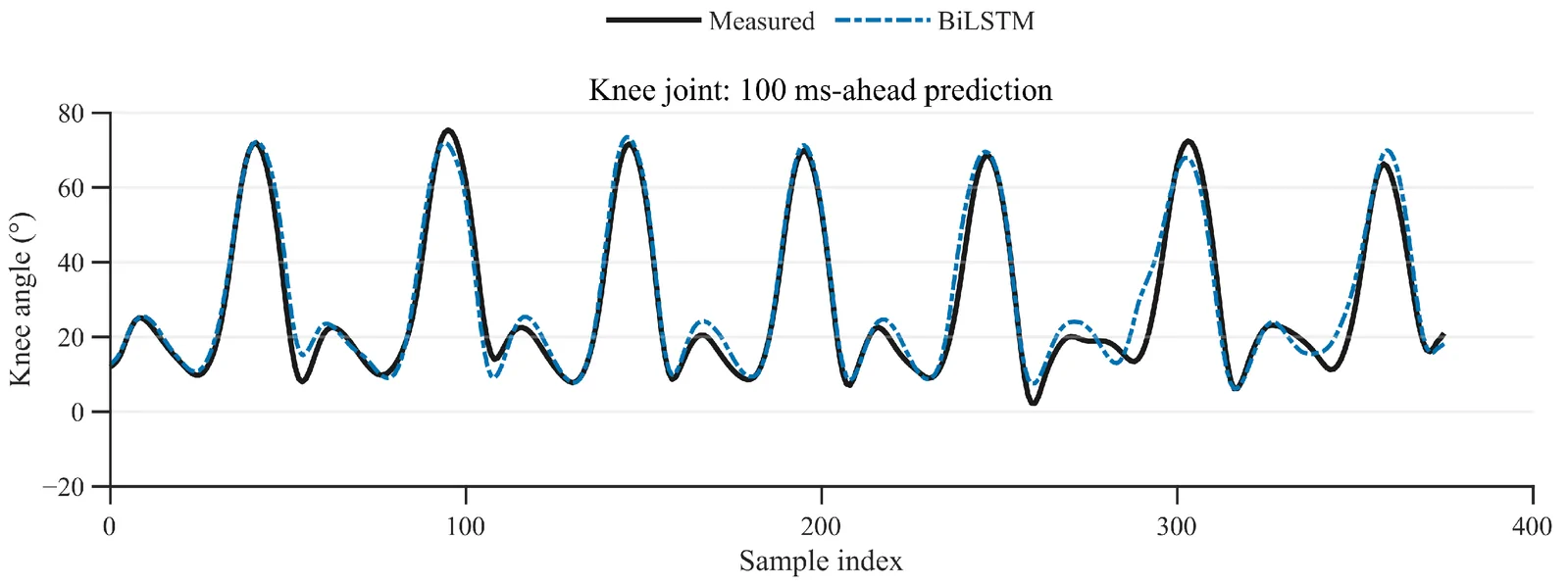
Illustrative BiLSTM prediction and IMU-derived measured knee angle over the fixed test-output interval defined at the beginning of Section 3.

**Figure 14:**
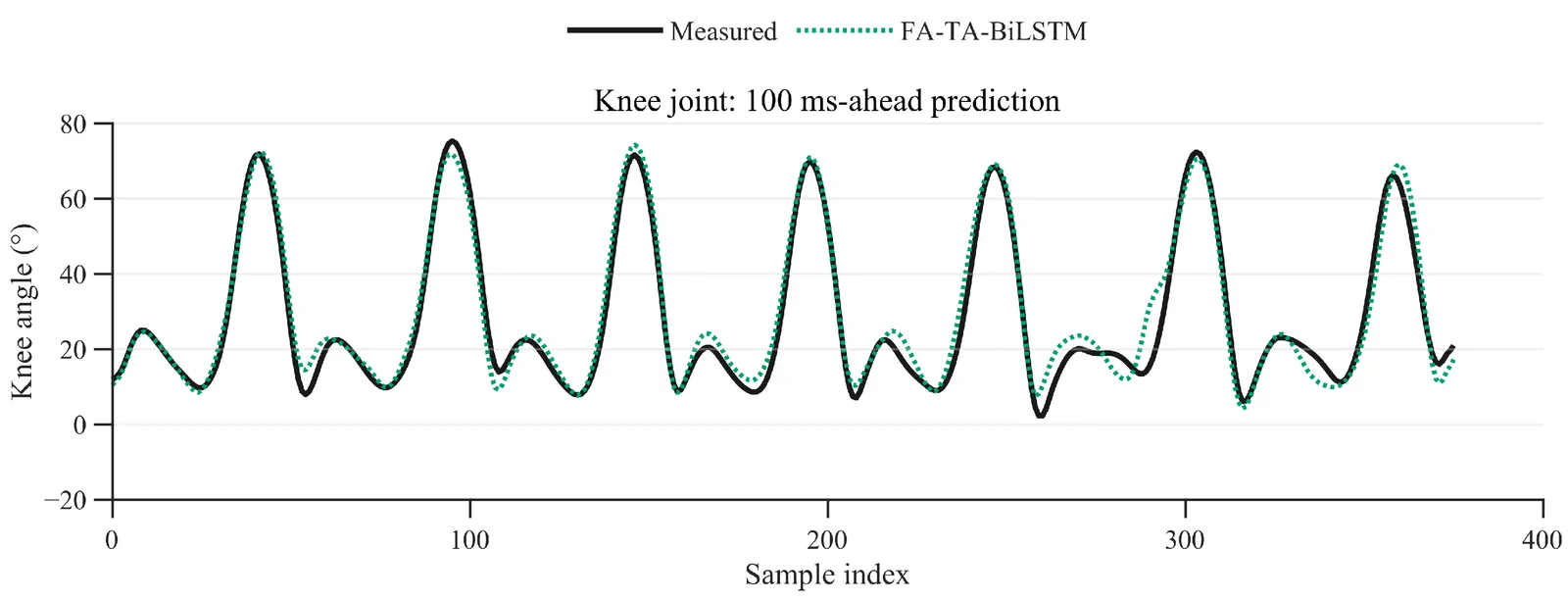
Illustrative FA-TA-BiLSTM prediction and IMU-derived measured knee angle over the fixed test-output interval defined at the beginning of Section 3.

**Figure 15:**
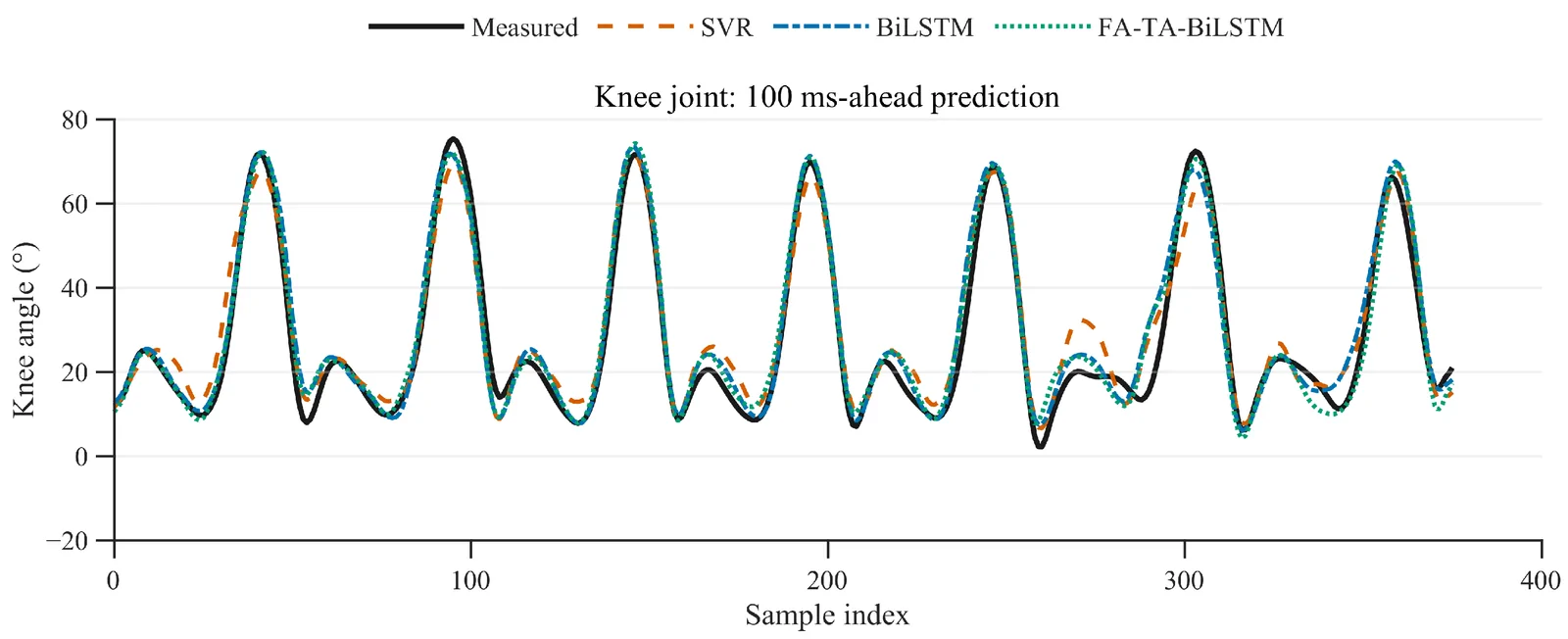
Illustrative comparison of knee angle predictions from the three models against the IMU-derived reference over the fixed test-output interval.

**Figure 16:**
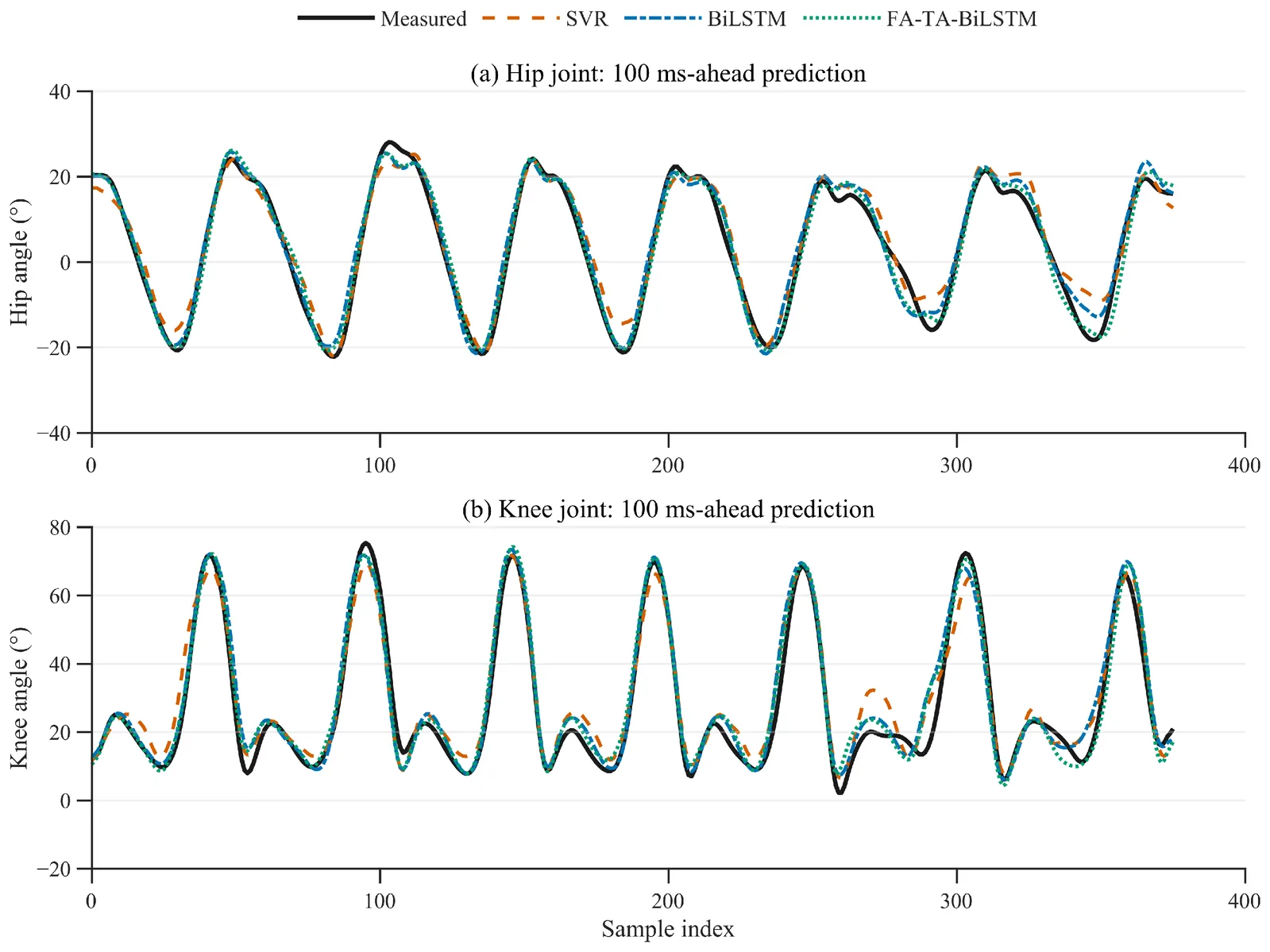
Illustrative overall comparison of hip and knee angle predictions from the three models against the IMU-derived references over the fixed test-output interval.

After model training, predictions were generated for the concatenated mixed-participant test set. Figure 8, Figure 9, Figure 10, Figure 11, Figure 12, Figure 13, Figure 14, Figure 15 and Figure 16 show the same fixed consecutive sample-index interval from all model outputs. The interval was selected for qualitative comparison without reference to prediction errors; because the archived plotting record did not retain participant, task, trial, or original-time identifiers, it is not presented as a participant-specific or uninterrupted-trial example. All quantitative metrics were calculated on the complete test set. MATLAB smoothdata was applied only to the displayed prediction curves and did not affect preprocessing, training, prediction generation, or the reported metrics.

### 3.1. Hip Joint Angle Prediction Results

The hip joint contributes to postural regulation, propulsion, and center-of-mass transfer during lower-limb movement. To evaluate short-horizon hip angle prediction, the outputs of SVR, BiLSTM, and FA-TA-BiLSTM were compared with the corresponding IMU-derived measurements.

Figure 8, Figure 9 and Figure 10 show illustrative hip angle predictions from SVR, BiLSTM, and FA-TA-BiLSTM, respectively. All three models follow the overall temporal pattern, but SVR shows larger local deviations, BiLSTM shows closer temporal agreement, and FA-TA-BiLSTM provides the closest visual agreement in most displayed cycles. Because no attention-module ablation was performed, this comparison concerns the complete architectures rather than either attention module separately.

Figure 11 provides a direct comparison and shows the same qualitative ordering among the three models.

Table 9 gives the quantitative evaluation results of different models in the hip joint angle prediction task. The RMSE, MAE, and $R^{2}$ of the SVR model are 3.7476, 2.8765, and 0.8823, respectively. The RMSE and MAE of the BiLSTM model decrease to 2.4798 and 2.0394, respectively, while $R^{2}$ increases to 0.9358. The FA-TA-BiLSTM model further reduces RMSE and MAE to 2.0684 and 1.5920, respectively, and increases $R^{2}$ to 0.9726. Compared with the SVR model, the FA-TA-BiLSTM model reduces RMSE by 44.80% and MAE by 44.65%. Compared with the BiLSTM model, the FA-TA-BiLSTM model reduces RMSE by 16.59% and MAE by 21.94%. These values are aggregate point estimates calculated on the current offline test samples; they are not intended to represent participant-level variability or cross-participant robustness.

### 3.2. Knee Joint Angle Prediction Results

The knee joint contributes to load acceptance, swing-phase control, and postural transitions. In the present dataset, the knee angle exhibits larger excursions, sharper local peaks, and faster changes than the hip angle, making short-horizon prediction more challenging.

Figure 12, Figure 13 and Figure 14 show illustrative knee angle predictions from SVR, BiLSTM, and FA-TA-BiLSTM, respectively. SVR shows larger deviations during rapid changes and near peaks, BiLSTM follows the measured curve more closely, and FA-TA-BiLSTM shows the closest overall visual agreement. These observations do not identify the separate contribution of either attention module.

Figure 15 compares the three knee predictions directly. The larger excursions and sharper peaks make knee prediction more difficult than hip prediction; SVR shows the largest local deviations, while BiLSTM and especially FA-TA-BiLSTM follow the measured curve more closely.

Table 10 lists the quantitative evaluation results of different models in the knee joint angle prediction task. The RMSE, MAE, and $R^{2}$ of the SVR model are 5.5673, 4.5126, and 0.8935, respectively. The RMSE and MAE of the BiLSTM model decrease to 3.9962 and 3.1512, respectively, while $R^{2}$ increases to 0.9357. The RMSE and MAE of the FA-TA-BiLSTM model further decrease to 2.9604 and 2.5142, respectively, while $R^{2}$ increases to 0.9660. Compared with the SVR model, the FA-TA-BiLSTM model reduces RMSE by 46.83% and MAE by 44.29%. Compared with the BiLSTM model, the FA-TA-BiLSTM model reduces RMSE by 25.92% and MAE by 20.21%. These results describe the aggregate performance on the current offline test samples and should be interpreted together with the limited participant sample size and the descriptive participant-level variability reported below.

### 3.3. Overall Comparison of Different Models

The aggregate metrics show the same ordering for both joints: SVR has the highest errors, BiLSTM has lower errors, and FA-TA-BiLSTM has the lowest errors. This pattern is consistent with recurrent temporal encoding and, in the complete FA-TA-BiLSTM architecture, the reweighting of input dimensions and historical states. Because module ablation and capacity matching were not performed, the gains cannot be attributed solely to the attention modules.

Table 11 reports the absolute errors and relative reductions in FA-TA-BiLSTM compared with the two baselines.

Table 12 reports the per-participant RMSE and MAE values for hip and knee joint prediction across the three compared models.

The mean and standard-deviation rows were calculated across the five participant-level values using the sample standard deviation. They may differ slightly from aggregate metrics because the numbers of test samples differed among participants. Participant P3 showed consistently higher errors than the other participants. This may reflect differences in movement patterns, joint angle ranges, task execution, or sensor–body interface conditions; these factors were not independently quantified, so no causal conclusion is drawn.

For hip RMSE, hip MAE, knee RMSE, and knee MAE, the model ranking was identical across all five participants. The Friedman result was therefore the same for each outcome ($\chi^{2}\left(2\right)=10.000$, p=0.0067, Kendall’s W=1.000), indicating complete rank concordance within this small cohort. However, the planned exact two-sided Wilcoxon comparisons of FA-TA-BiLSTM with either SVR or standard BiLSTM yielded p=0.0625 in each case; after Holm adjustment within each outcome, the adjusted value was p=0.1250. Thus, the participant-level differences were directionally consistent but did not provide confirmatory pairwise significance at the 0.05 level.

Figure 16 summarizes the illustrative hip and knee comparisons; together with the aggregate metrics, it shows that FA-TA-BiLSTM performs favorably relative to the selected baselines on this dataset.

### 3.4. Analysis of Prediction Errors

Errors are most apparent near extrema, direction reversals, and rapid flexion–extension. All models show higher knee than hip errors, consistent with the knee’s larger excursions and faster local changes. SVR has the largest local deviations, whereas FA-TA-BiLSTM has the lowest aggregate RMSE and MAE for both joints; these visual observations remain qualitative and should be interpreted with the complete-set metrics.

## 4. Discussion

### 4.1. Prediction Performance of the FA-TA-BiLSTM Model

Under the same combined-input condition and prediction setting, FA-TA-BiLSTM produced lower errors and higher goodness-of-fit than SVR and standard BiLSTM for both joints. The complete architecture combines feature-wise input reweighting, bidirectional recurrent encoding, and temporal-attention aggregation. Because no attention-module ablation was performed, and model capacity was not matched between FA-TA-BiLSTM and standard BiLSTM, the observed difference cannot be attributed exclusively to the attention design.

The hip and knee RMSE values were 2.07° and 2.96°, respectively. Coker et al. reported 2.12 ± 0.69° for 100 ms-ahead knee angle prediction using EMG and historical knee angles, while Li et al. reported 4.81 ± 1.37° for sEMG-based knee angle estimation [7,36]. The present errors are of a similar order of magnitude to those reported in these related offline studies. However, differences in tasks, inputs, reference measurements, and validation protocols preclude a universal clinical or control-level acceptability threshold.

### 4.2. Role of Multi-Source Inputs

At the 100 ms horizon, historical joint angles and angular velocities provide direct autoregressive cues about motion state and trend, whereas sEMG provides complementary physiological information. The kinematic inputs are not the target angle at the prediction time: they are measurements available before the prediction origin, and the target is the joint state 100 ms later. The practical purpose is therefore sensor-based short-horizon forecasting, not reconstruction of kinematics solely from biosignals. This formulation is relevant to systems in which joint-state sensors are already available and a future-state estimate may assist delay compensation or anticipatory control. Because no modality-ablation experiment was performed, the current comparison evaluates the complete combined-input model and does not establish the incremental contribution of sEMG beyond the kinematic inputs.

### 4.3. Application Scope of the Offline Prediction Method

This work evaluates a unified 100 ms-ahead framework that combines a 36-dimensional six-channel sEMG representation with pre-target hip and knee angles and angular velocities, applies feature and temporal attention, and predicts both future joint angles. Because historical kinematics are retained as autoregressive inputs, the task is short-horizon state forecasting rather than biosignal-only decoding. The comparison does not establish independent modality or module effects, superiority over untested advanced models, real-time control performance, or rehabilitation efficacy.

### 4.4. Limitations and Future Work

The cohort comprised only five healthy adult men. Each participant was divided chronologically into training, validation, and test subsets before the corresponding subsets were pooled across participants. Thus, every participant contributed data to all three subsets, while 75% overlap between adjacent windows may leave correlation near subset boundaries. The reported values are therefore preliminary within-cohort estimates rather than evidence of participant-independent generalization. Larger sex-, age-, and pathology-diverse cohorts and participant-independent validation are required.

The comparison was restricted to SVR, BiLSTM, and the complete FA-TA-BiLSTM model. No TCN or Transformer benchmark, modality or attention-module ablation, feature-selection analysis, or multiple-horizon evaluation was performed. Correlated sEMG features and unmatched model capacity also limit attribution of the observed improvement. Future work should separate modality, module, feature-set, model-capacity, and prediction-horizon effects.

The prediction references were IMU-derived angles without independent optical or goniometric validation. Timestamp alignment, cubic-spline resampling, and independent device clocks may introduce residual measurement or timing uncertainty. Data from level walking and sit-to-stand transitions were pooled, so task-specific performance was not quantified. Because recordings were concatenated before sliding-window construction, some samples near recording or task junctions may combine adjacent segments, and the influence of these boundary samples was not independently quantified. In addition, the buffered BiLSTM architecture is not directly equivalent to causal online streaming and should be compared with unidirectional or other causal models before real-time deployment.

The sample size was feasibility-driven; no a priori power analysis was performed, and only five participants were available for participant-level inference. Exploratory Friedman tests indicated fully consistent model rankings across participants, but the planned exact pairwise Wilcoxon comparisons did not reach the 0.05 level after Holm adjustment. The numerical differences should therefore not be interpreted as confirmatory evidence of population-level superiority.

## 5. Conclusions

This preliminary offline study used data from five healthy adult men and a mixed-participant chronological split; the findings describe within-cohort performance and should not be generalized to unseen participants, clinical populations, or real-time control.

Under the same combined-input condition and 100 ms horizon, FA-TA-BiLSTM produced lower aggregate errors and higher $R^{2}$ values than SVR and standard BiLSTM. However, exact participant-level pairwise comparisons did not reach significance after Holm adjustment, so the results support feasibility on the current dataset rather than population-level superiority or isolated modality and module effects. Future work should include larger and more diverse cohorts, participant-independent validation, ablation, task-specific analysis, independently validated angle references, multiple horizons, and causal online implementation. 

## Figures and Tables

**Table 1:** Basic information of the participants.

No.	Sex	Age, Years	Height, cm	Weight, kg	BMI, kg/m^2^	Self-Reported Condition
1	Male	27	176	70	22.60	No reported relevant condition
2	Male	25	174	71	23.45	No reported relevant condition
3	Male	24	169	69	24.16	No reported relevant condition
4	Male	25	180	80	24.69	No reported relevant condition
5	Male	24	183	73	21.80	No reported relevant condition

**Table 2:** Functional roles of multi-source motion information.

Information Type	Meaning	Main Role	Model Usage
sEMG signal	Electrical activity related to muscle activation	Reflects voluntary motion intention and muscle coordination	Provides neuromuscular features for prediction
Joint angle	Current posture of the hip and knee joints	Describes lower-limb kinematic state	Provides state information available before prediction
Joint angular velocity	Rate of change of joint angle	Describes motion trend and transition process	Provides dynamic information for short-horizon forecasting

**Table 3:** Functional description of target muscles.

Muscle Abbreviation	Muscle Name	Main Function	Corresponding Motion Role
Gmax	Gluteus maximus	Hip extension	Reflects hip extension and support propulsion
Gmed	Gluteus medius	Hip stabilization and pelvic control	Reflects pelvic stability and weight transfer
RF	Rectus femoris	Hip flexion and knee extension	Represents hip–knee coordinated activity
VL	Vastus lateralis	Knee extension	Reflects knee extensor output
BF	Biceps femoris	Knee flexion and auxiliary hip extension	Reflects posterior muscle braking and knee flexion
TA	Tibialis anterior	Ankle dorsiflexion	Reflects foot control and gait-timing information

**Table 4:** sEMG feature definitions used in this study.

Feature	Definition	Main Meaning
IEMG	$\sum_{k=1}^{N}\left|x\left(k\right)\right|$	Accumulated muscle activation amplitude
RMS	$\sqrt{\frac{1}{N}\sum_{k=1}^{N}x^{2}\left(k\right)}$	Signal energy level
WL	$\sum_{k=1}^{N-1}\left|x\left(k+1\right)-x\left(k\right)\right|$	Waveform variation complexity
DASDV	$\sqrt{\frac{1}{N-1}\sum_{k=1}^{N-1}{\left[x(k+1)-x(k)\right]}^{2}}$	Local fluctuation intensity
MF	$\sum_{f=0}^{f_{\mathrm{MF}}}P\left(f\right)=\frac{1}{2}\sum_{f=0}^{f_{\max}}P\left(f\right)$	Frequency dividing the total spectral power into two equal parts
SE	$-\sum_{j=1}^{M}p_{j}\log\left(p_{j}\right)$	Entropy of the normalized power spectrum

**Table 5:** Construction of the multi-source input vector.

Input Component	Feature Description	Dimension
sEMG features	IEMG, RMS, WL, DASDV, MF, and SE extracted from six sEMG channels	6×6=36
Joint angles	Synchronized hip and knee joint angles	2
Joint angular velocities	Hip and knee joint angular velocities calculated from synchronized angles	2
Multi-source input vector	Concatenation of sEMG features, joint angles, and joint angular velocities	40

**Table 6:** Configuration of the short-horizon prediction task.

Item	Setting
Prediction target	Future hip and knee joint angles after the current feature window
Prediction horizon	100 ms
sEMG sampling frequency	1000 Hz
IMU sampling frequency	200 Hz
Input sequence	Current and historical multi-source sequence, including sEMG features, joint angles, and angular velocities
Sliding window length	200 ms
Window step	50 ms
Input sequence length	16 consecutive feature windows
Approximate historical span	950 ms from the beginning of the first window to the end of the last window
Label shift	Two feature steps relative to the last input window
Dataset division	Within-participant chronological split into 70% training, 15% validation, and 15% test subsets, followed by pooling the corresponding subsets across participants; no participant-independent split

**Table 7:** Dataset size and participant-specific chronological split after concatenation and window construction.

Participant	Recording Duration, s	Usable Labeled Windows	Sequence Samples	Training Samples	Validation Samples	Test Samples
P1	785.356	15,702	15,687	10,980	2353	2354
P2	782.372	15,642	15,627	10,938	2344	2345
P3	800.830	16,011	15,996	11,197	2399	2400
P4	701.312	14,021	14,006	9804	2100	2102
P5	663.701	13,269	13,254	9277	1988	1989
Total	3733.571	74,645	74,570	52,196	11,184	11,190

**Table 8:** Input Configurations, principal model components, and training settings of the compared models.

Model	Input Configuration and Principal Model Components	Training Settings
SVR	The 40-dimensional historical sequence was flattened; Gaussian kernel; KernelScale = auto; BoxConstraint = 30; Epsilon = 0.06; Standardize = false. Separate regressors were trained for hip and knee joint angles.	The principal SVR parameters were manually selected during preliminary development with reference to validation-set performance.
BiLSTM	A 40-dimensional historical input sequence; one BiLSTM temporal encoder with 40 hidden units and OutputMode = last; dropout = 0.45; a fully connected layer with 20 units; ReLU; a two-output regression layer.	Adam optimizer; 26 epochs; batch size = 128; initial learning rate = 0.001; gradient threshold = 1; data shuffled every epoch.
FA-TA-BiLSTM	A 40-dimensional historical input sequence; feature-attention weighting; stacked BiLSTM temporal encoding with principal hidden-unit settings of 128 and 96; temporal-attention aggregation; dropout = 0.08; fully connected layer with 72 units; ReLU; two-output regression layer.	Adam optimizer; 70 epochs; batch size = 128; initial learning rate = 0.0006; gradient threshold = 1; data shuffled every epoch. Hyperparameters were manually selected with reference to validation performance and training stability; no exhaustive grid or random search was conducted.

**Table 9:** Performance comparison for hip joint angle prediction.

Model	RMSE, °	MAE, °	$R^{2}$
SVR	3.7476	2.8765	0.8823
BiLSTM	2.4798	2.0394	0.9358
FA-TA-BiLSTM	2.0684	1.5920	0.9726

**Table 10:** Performance comparison for knee joint angle prediction.

Model	RMSE, °	MAE, °	$R^{2}$
SVR	5.5673	4.5126	0.8935
BiLSTM	3.9962	3.1512	0.9357
FA-TA-BiLSTM	2.9604	2.5142	0.9660

**Table 11:** Absolute error values and relative error reductions in FA-TA-BiLSTM compared with baseline models in the short-horizon prediction task.

Joint	Baseline	Baseline RMSE, °	FA-TA RMSE, °	RMSE Reduction, %	Baseline MAE, °	FA-TA MAE, °	MAE Reduction, %
Hip	SVR	3.7476	2.0684	44.80	2.8765	1.5920	44.65
Hip	BiLSTM	2.4798	2.0684	16.59	2.0394	1.5920	21.94
Knee	SVR	5.5673	2.9604	46.83	4.5126	2.5142	44.29
Knee	BiLSTM	3.9962	2.9604	25.92	3.1512	2.5142	20.21

**Table 12:** Per-participant RMSE and MAE for hip and knee joint angle prediction.

Participant	Model	Hip RMSE, °	Hip MAE, °	Knee RMSE, °	Knee MAE, °
P1	SVR	3.6041	2.7923	5.3793	4.3635
P1	BiLSTM	2.3882	1.9684	3.9325	3.1261
P1	FA-TA-BiLSTM	1.9877	1.5388	2.8691	2.4184
P2	SVR	3.5792	2.7948	5.7280	4.6432
P2	BiLSTM	2.3487	1.9476	3.7527	2.9631
P2	FA-TA-BiLSTM	1.9524	1.4970	2.7684	2.3641
P3	SVR	4.2680	3.2515	6.3404	5.1009
P3	BiLSTM	2.8042	2.3053	4.5111	3.5620
P3	FA-TA-BiLSTM	2.3456	1.7995	3.3815	2.8419
P4	SVR	3.6690	2.8486	5.4505	4.4689
P4	BiLSTM	2.4378	2.0196	3.9324	3.1207
P4	FA-TA-BiLSTM	2.0150	1.5866	2.8783	2.4898
P5	SVR	3.5941	2.7911	5.3393	4.3786
P5	BiLSTM	2.3882	1.9788	3.8525	3.0576
P5	FA-TA-BiLSTM	1.9937	1.5647	2.8491	2.4395
Mean ± SD	SVR	3.7429 ± 0.2955	2.8957 ± 0.2004	5.6475 ± 0.4160	4.5910 ± 0.3060
Mean ± SD	BiLSTM	2.4734 ± 0.1876	2.0439 ± 0.1484	3.9962 ± 0.2971	3.1659 ± 0.2310
Mean ± SD	FA-TA-BiLSTM	2.0589 ± 0.1619	1.5973 ± 0.1178	2.9493 ± 0.2455	2.5107 ± 0.1905

## Data Availability

The data presented in this study are available from the corresponding author upon reasonable request. The data are not publicly available due to privacy considerations related to human-participant recordings.

## Abbreviations

The following abbreviations are used in this manuscript:

sEMG	Surface electromyography
IMU	Inertial measurement unit
SVR	Support vector regression
LSTM	Long short-term memory
BiLSTM	Bidirectional long short-term memory
FA	Feature attention
TA	Temporal attention
FA-TA-BiLSTM	Feature-attention and temporal-attention bidirectional long short-term memory
IEMG	Integrated electromyography
RMS	Root mean square
WL	Waveform length
DASDV	Difference absolute standard deviation value
MF	Median frequency
SE	Spectral entropy
RMSE	Root mean square error
MAE	Mean absolute error
$R^{2}$	Coefficient of determination
